# Using Grizzly Bears to Assess Harvest-Ecosystem Tradeoffs in Salmon Fisheries

**DOI:** 10.1371/journal.pbio.1001303

**Published:** 2012-04-10

**Authors:** Taal Levi, Chris T. Darimont, Misty MacDuffee, Marc Mangel, Paul Paquet, Christopher C. Wilmers

**Affiliations:** 1 Environmental Studies Department, University of California, Santa Cruz, California, United States of America; 2 Center for Stock Assessment Research, Department of Applied Math and Statistics, University of California, Santa Cruz, California, United States of America; 3 Raincoast Conservation Foundation, Denny Island, British Columbia, Canada; 4 Department of Biology, University of Bergen, Bergen, Norway; 5 Faculty of Environmental Design, University of Calgary, Calgary, Alberta, Canada; Cornell University, United States of America

## Abstract

Implementation of ecosystem-based fisheries management (EBFM) requires a clear conceptual and quantitative framework for assessing how different harvest options can modify benefits to ecosystem and human beneficiaries. We address this social-ecological need for Pacific salmon fisheries, which are economically valuable but intercept much of the annual pulse of nutrient subsidies that salmon provide to terrestrial and aquatic food webs. We used grizzly bears, vectors of salmon nutrients and animals with densities strongly coupled to salmon abundance, as surrogates for “salmon ecosystem” function. Combining salmon biomass and stock-recruitment data with stable isotope analysis, we assess potential tradeoffs between fishery yields and bear population densities for six sockeye salmon stocks in Bristol Bay, Alaska, and British Columbia (BC), Canada. For the coastal stocks, we find that both bear densities and fishery yields would increase substantially if ecosystem allocations of salmon increase from currently applied lower to upper goals and beyond. This aligning of benefits comes at a potential cost, however, with the possibility of forgoing harvests in low productivity years. In contrast, we detect acute tradeoffs between bear densities and fishery yields in interior stocks within the Fraser River, BC, where biomass from other salmon species is low. There, increasing salmon allocations to ecosystems would benefit threatened bear populations at the cost of reduced long-term yields. To resolve this conflict, we propose an EBFM goal that values fisheries and bears (and by extension, the ecosystem) equally. At such targets, ecosystem benefits are unexpectedly large compared with losses in fishery yields. To explore other management options, we generate tradeoff curves that provide stock-specific accounting of the expected loss to fishers and gain to bears as more salmon escape the fishery. Our approach, modified to suit multiple scenarios, provides a generalizable method to resolve conflicts over shared resources in other systems.

## Abstract 2

Using grizzly bears as surrogates for “salmon ecosystem” function, the authors develop a generalizable ecosystem-based management framework that enables decision-makers to quantify ecosystem-harvest tradeoffs between wild and human recipients of natural resources like fish.

## Author Summary

Commercial fisheries that harvest salmon for human consumption can end up diverting nutrients that would normally be directed to terrestrial and aquatic ecosystems. We examined this problem for Pacific salmon fisheries by using grizzly bears as indicators of salmon ecosystem function. Bear densities vary enormously depending on salmon availability, and by leaving uneaten salmon carcass remains beside spawning streams, bears play an important role in dispersing marine nutrients to plants, invertebrates, and other wildlife. By relating the number of spawning fish to bear diet and density, we developed a model to quantify “ecosystem-harvest” tradeoffs; i.e., how bear density changes with the amount of fish harvested (fishery yields). We estimated this tradeoff between yields and bear density for six sockeye salmon stocks in Alaska and British Columbia (BC) across a range of management options that varied the number of salmon allowed to escape from the fishery. Our model shows that bear densities will increase substantially with more spawning fish at all sites. Notably, in most study systems, fishery yields are also expected to increase as the number of spawning fish increases. There is one exception, however, in the Fraser River (BC), where bears are threatened and sockeye salmon are nearly the only species of salmon available. Here, releasing more salmon to spawn would result in lower fishery yields. To resolve such conflicts in this and other systems, we propose a generalizable ecosystem-based fisheries management framework, which allows decision-makers (such as fisheries managers and conservation scientists) to evaluate different allocation options between fisheries and other ecosystem recipients.

## Introduction

Due to the impacts of fisheries on non-target species and ecological processes, there is growing pressure to apply ecosystem-based fisheries management (EBFM) [1]–[4]. Guiding principles exist, but EBFM cannot be implemented without quantitative methods that can guide policy. Additionally, designing EBFM approaches requires an assessment of the tradeoffs inherent to balancing ecosystem protection and economic costs. This is because any EBFM plan, however technically robust, requires political will. Confronting these challenges requires a new focus on case studies that account for the unique biology of each fishery and from which general guidance might emerge for other systems.

Pacific salmon (*Oncorhynchus* spp.) are economically, socio-culturally, and ecologically important. Alaskan landings alone surpass 300,000 metric tons and ex-vessel values exceed US$260 million annually [5]. Many cultures, aboriginal and otherwise, are also tied to salmon [6]. Transcending value to humans, adult wild salmon are critical to aquatic, terrestrial, and marine ecosystem function. They are the dominant prey of a number of marine and terrestrial predators such as orcas [7], salmon sharks [8], pinnipeds [9], and grizzly bears [10]. Salmon carcasses, distributed primarily by bears during spawning events, contribute annual pulses of marine-derived nutrients to freshwater systems that propagate through food webs and influence primary producers, invertebrates, fish, and wildlife [11].

The inherent conflict between the socio-economic value of salmon and their critical role in ecosystem function has led to calls for a change from current single-species management to EBFM [12]. However, such challenges have yet to lead to scientifically grounded and quantitative policy recommendations that can inform managers and fishery certifiers such as the Marine Stewardship Council (MSC). One of the MSC's guiding principles is that fisheries must minimize ecosystem impacts, but it remains unclear how to quantify i) the impact that competition with fisheries has on wildlife, ii) the influence of modifying harvest levels on the ecosystem, iii) or the economic costs of various management options.

Selecting which organisms to monitor is also a consistent problem in the implementation of EBFM because knowledge of the relationships between biomass availability of the central resource and population responses of non-human consumers are often limited [13]–[15]. Here we cross ecosystem boundaries to use a terrestrial animal, the grizzly bear (*Ursus arctos horribilis*), as a focal species to develop a quantitative framework that evaluates the tradeoffs between fisheries yields and an ecosystem response to salmon (i.e., grizzly bear densities).

**Figure 1:**
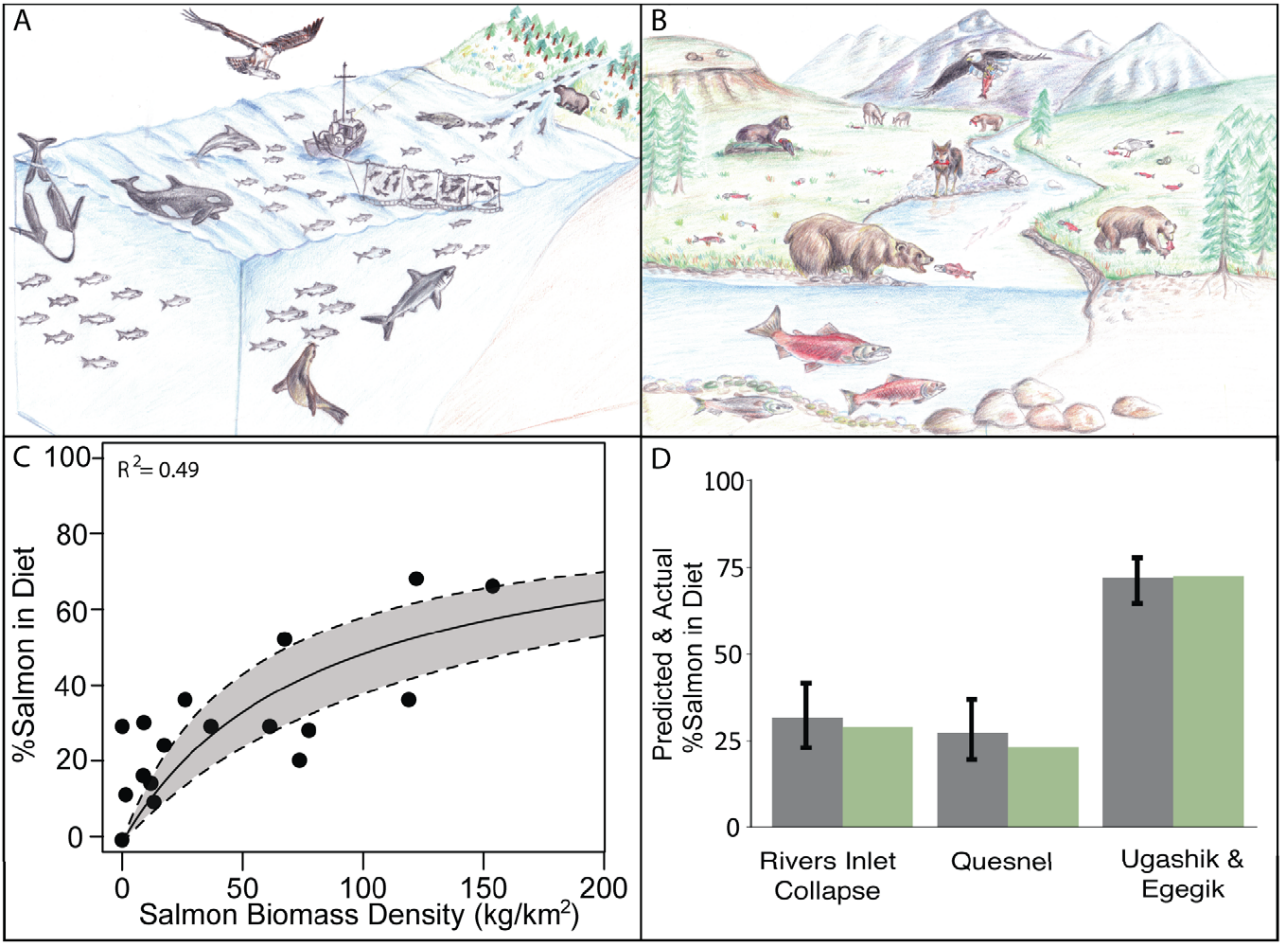
Using bears to quantify the importance of salmon to wildlife. Mature salmon are (A) important prey to orcas, pinnipeds, salmon sharks, humans, and other predators in the marine domain before they (B) reach terrestrial and aquatic systems, where they supply annual pulses of marine-derived nutrients and are the dominant prey of grizzly bears. By leaving uneaten carcass remains in riparian areas, bears serve as vectors of salmon to terrestrial and aquatic systems, supplying nutrients and food to riparian vegetation, invertebrates, and vertebrate scavengers including canids, gulls, eagles, and mustelids. The importance of salmon to bears can be quantified with (C) the relationship between salmon density and salmon consumption by bears as determined by stable isotope analysis of 18 grizzly bear populations from British Columbia (BC) [36]. (D) Predicted salmon consumption by bears (gray bars with 95% confidence intervals) closely matches measured salmon consumption (green bars) as estimated by stable isotope analysis in bears from Rivers Inlet and Quesnel Lake in interior BC, and for the Ugashik and Egegik stocks combined in Bristol Bay, Alaska.

We chose grizzly bears, which are also called brown bears in coastal systems, as a surrogate of salmon-influenced ecosystem function because 1) bear population dynamics are strongly linked to salmon abundance [10]; 2) bears are the *terminal* predator, consuming salmon in their final life history phase; thus, if there are enough salmon to sustain healthy bear densities, we reason that there should be sufficient salmon numbers to sustain populations of earlier salmon-life-history predators such as seabirds, pinnipeds, and sharks (Figure 1A and 1B); and 3) bears are the dominant species mediating the flow of salmon-derived nutrients from the ocean to the terrestrial ecosystem (Figure 1B) [16]. After capturing salmon in estuaries and streams, grizzly bears typically move to land to consume each fish, distributing carcass remains to vertebrate and invertebrate scavengers up to several hundred meters from waterways [17],[18]. Carcass remains (nutrients and energy) can influence all trophic levels from primary producers to large carnivores in both terrestrial and aquatic ecosystems [16],[19],[20]. Described as a “keystone interaction”, this coupled grizzly-salmon association (at high bear densities) can provide up to a quarter of the nitrogen budget to plant communities in riparian areas adjacent to spawning grounds [19]. Additional benefits provided by a focus on grizzly bears are their charismatic appeal to the public and their status as a large carnivore commonly of conservation concern.

The fundamental challenge with implementing EBFM in this bear-salmon-human system (and others) is to determine how much of the fished resource to allocate to fisheries versus the ecosystem. Currently, under single-species management, fisheries commonly intercept more than 50% of inbound salmon that would otherwise be available to bears and the terrestrial and aquatic ecosystems they support [6]. Managers, typically focused exclusively on prioritizing allocation to fisheries, determine an optimum number of the total salmon run to allocate to spawning, or “escapement”. The goal is generally to achieve maximum sustainable yield (MSY), but the political process, uncertainty in the relationship between spawning stock (escapement) and recruitment, and multiple management objectives can result in escapement goals below an estimated MSY level (see below). For fisheries like this, managed below MSY, both yield and bear density would increase with greater escapement, but the potential responses have not been explored quantitatively. For those managed at MSY, increased escapement would benefit grizzly bears (and the ecosystem), but costs would be borne by fishers via losses in yield. The precise tradeoffs, however, require a detailed quantitative assessment over a range of managed escapements to be of maximum value to decision-makers faced with this potentially contentious change to salmon management.

**Figure 2:**
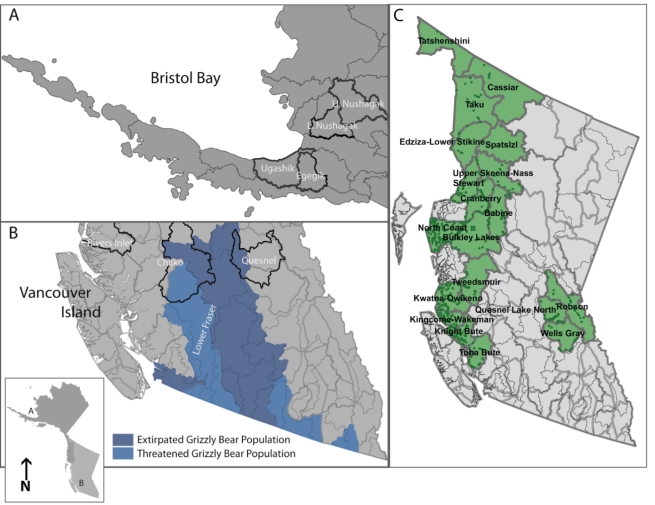
Salmon stocks and grizzly bear population units (GBPUs) used in our analyses. We consider three sockeye salmon stocks from (A) Bristol Bay, Alaska, and (B) two stocks from the Fraser River, British Columbia (BC), Canada, and one from the mid-coast of BC (Rivers Inlet). Watersheds are outlined by thin gray lines and focal watersheds are outlined in black. In BC, thick gray lines denote designated GBPUs from which isotope data were derived. The Chilko and Quesnel stocks are in a region of bear conservation concern. (C) Percent salmon in grizzly bear diet as a function of salmon availability across 18 GBPUs in BC. Stable isotope data were collected from 1995 to 2003 in green-filled GBPUs. We first allocated mean salmon biomass measured at points from 1995 to 2003 to watersheds (thin lines). We then allocated salmon biomass to grizzly GBPUs (thick lines) based on the area of intersection between watersheds and GBPUs.

**Figure 3:**
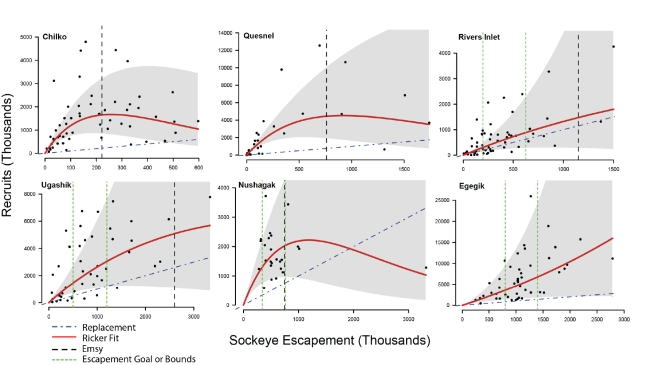
Stock recruitment relationships for study systems, fit with the Ricker stock-recruitment model. The difference between recruitment and the replacement line is considered surplus production that can be sustainably harvested. This difference is maximized at *E_MSY_*, but the lower and upper target escapements are often well below estimates of *E_MSY_*. The escapement in the absence of the fishery, *E_m_*, is estimated at the steady state of the Ricker model, which is best visualized at the intersection of the Ricker and replacement lines.

To evaluate the effects of different management options, we modeled how bear population densities and fisheries yields would respond to increased escapement. This involved first estimating a relationship between salmon biomass availability and salmon consumption by bears from 18 grizzly bear populations across British Columbia (BC), Canada (Figure 1C and 1D). We linked this relationship to a known positive relationship between meat (i.e., salmon) consumption by grizzlies and grizzly densities [10],[21]. We then used stock-recruitment models, specific to sockeye salmon (*O. nerka*) stocks that spawn in Bristol Bay, Alaska, and BC (Figure 2), to estimate fisheries yields as a function of escapement, and the expected abundance of salmon in the absence of the fishery (Figure 3). For stocks managed below a MSY escapement, we assessed how departures from status quo management would increase bear densities and fisheries yields. For stocks managed at MSY, we scaled bear density and fishery yield by their system-specific maxima to create dimensionless and commensurate values that could be compared. In all assessments, we focused on sockeye while holding other salmonids at their management escapement targets, or mean escapement levels, because sockeye i) are often dominant runs, ii) migrate deep into interior regions, iii) are the most commercially valuable species [6], and iv) are species for which high quality stock-recruitment data exist.

While this work aims to develop a new conceptual and quantitative framework applicable to other resource management contexts, we also seek to inform contemporary bear and salmon management in BC and Alaska. First, we model potential population responses by grizzly bears in the Fraser River watershed, where bears are provincially threatened in the Chilko and partially extirpated in the Quesnel system (Figure 2). Second, we assess whether competition with the salmon fishery has the potential to significantly constrain grizzly bear productivity. This is particularly relevant because both the Fraser River and Bristol Bay stocks are certified by the MSC, having satisfied the minimal ecosystem impact principle.

## Results

In all systems, bear diets would respond considerably to increases in salmon abundance (i.e., escapement). Despite the myriad potential errors in estimating both variables across such large spatial scales, we found that salmon biomass availability alone explained nearly 50% of the variation in bear diets (% salmon in diet), which followed a saturating trend (Figure 1C). The relative accessibility of salmon that spawn in varied habitats, from small streams to rivers to lakeshores, likely explains some of the additional variability. Statistically fitting this relationship to 18 grizzly bear populations accounted for errors to produce a robust estimate of the relationship between salmon availability and salmon in bear diets. We estimated that the salmon biomass density necessary for salmon to constitute roughly 45% of bear diets (half of the recorded maximum salmon consumption by bears; see Materials and Methods) is 80.08 kg/km^2^, with a 95% confidence interval from 50.9 to 128.4 kg/km^2^. This population scale model was robust at other scales, accurately predicting bear diets at the watershed scale for three systems with known salmon biomass (Figure 1D; Table S1). This model, which predicts how percent salmon in bear diets responds to increased salmon escapements, helps explain corresponding increases in bear densities (see below; Materials and Methods).

Increased escapements relative to current management levels would also affect long-term fisheries yields, though patterns differ among systems. By fitting stock-recruitment relationships for each fishery, we identified three qualitatively distinct types of sockeye management dynamics (Figure 3). The Chilko and Quesnel stocks (Fraser River) exhibit clear overcompensating density dependence (when recruitment declines as the number of spawners increases). For these stocks, both the escapement that produces MSY, *E_MSY_*, and the escapement in the absence of a fishery, *E_m_*, could be reasonably estimated. These fisheries are currently managed at MSY (Figure 3). The Ugashik and Nushagak stocks are data poor in the upper regions of escapement, making *E_m_* difficult to estimate, but reasonable estimates of *E_MSY_* are possible. These systems are managed for lower and upper escapement goals, which are both below an estimated *E_MSY_*. Finally, the Egegik and Rivers Inlet stocks have the highest uncertainty because it is unclear if the stock-recruitment relationship is even appropriate to characterize the data. Recruitment in the Egegik stock does not saturate over the observed range of escapement, which is strong evidence that escapement goals could increase to reach *E_MSY_*. Similarly, management here occurs with lower and upper escapement goals, both below predicted *E_MSY_*. Rivers Inlet is uncertain because after a period of high productivity, the stock has collapsed and is slowly rebuilding, which raises the possibility that unobserved factors (e.g., changing productivity due to a regime shift) are driving recruitment dynamics [22]. Rather than consider upper and lower escapement goals for this stock in our analyses, we consider the escapement above which fishing is currently allowed and the optimal (and higher) escapement target estimated from a lake productivity model [22]. Although fishery yields are difficult to assess when there is high uncertainty in the stock-recruitment relationship, the impact of increasing escapement on bear densities can still be assessed.

**Figure 4:**
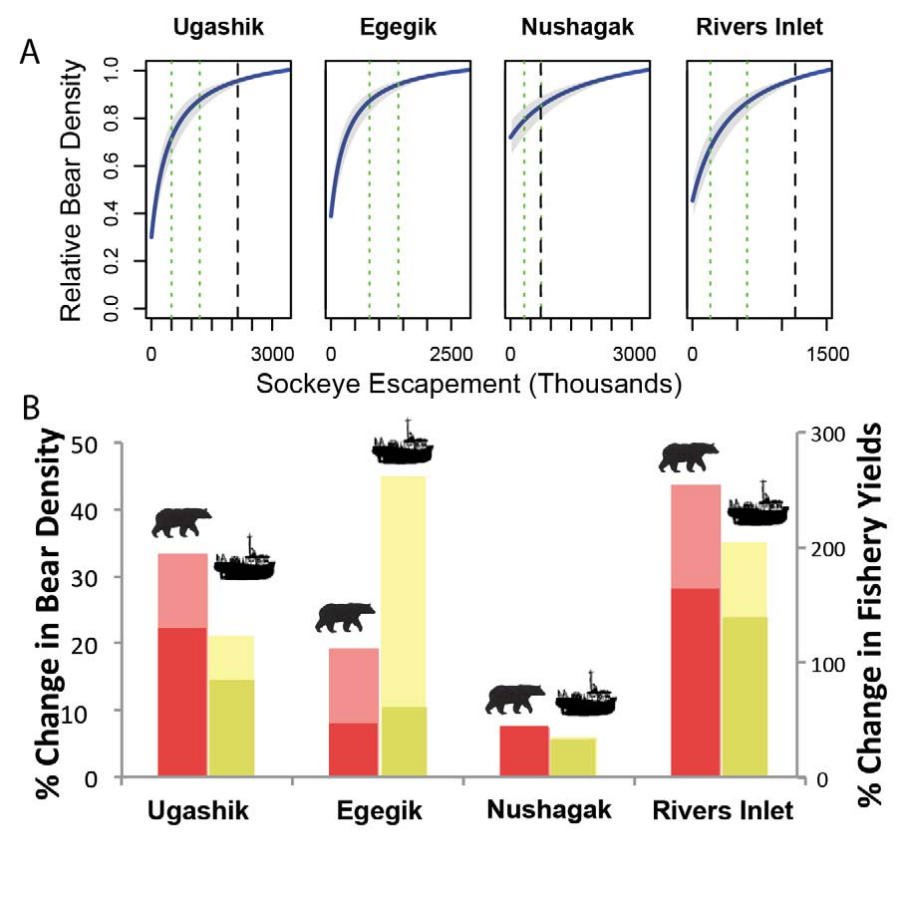
Accounting for bears when setting escapement goals in Bristol Bay and Rivers Inlet. (A) Bear density as a function of sockeye salmon escapement relative to the expected bear density at the maximum observed escapement (solid blue line). Vertical black dashed lines indicate *E_MSY_*. The lower and upper escapement goals are highlighted by green dotted lines. (B) Increasing escapements from the lower to upper goals can substantially increase bear density (lower dark-red bar). Further increases in escapement to *E_MSY_* continue to increase bear density (upper light-red bar), but the benefit is somewhat less due to the saturating relationship between escapement and percent salmon in diet. Importantly, there is no expected tradeoff to increasing escapement; yields are expected to be higher at upper escapement goals (lower dark-yellow bar) and increase further until *E_MSY_* (upper light-yellow bar). Although *E_MSY_* and the response in fisheries yields are uncertain, especially for the Egegik stock, bear success can still be assessed at the tangible lower and upper escapement goals and beyond.

We found that the presence and degree of conflict between fisheries yields and bear densities is stock-specific. Increasing escapement from lower to upper management targets in Rivers Inlet and the Alaskan systems would increase not only bear densities, but also fisheries yields (Figure 4A and 4B). Compared with the lower goals, the upper escapement goals of Ugashik, Egegik, Nushagak, and Rivers Inlet are expected to provide for roughly 22%, 8%, 8%, and 28% increases in bear density, respectively; if escapements were to increase from the lower goals to the estimated *E_MSY_* levels, bear density would increase by roughly 34%, 19%, 8%, and 44%, respectively (Figure 4B). Notably, expected increases in yield are proportionately much greater than increases in bear densities (Figure 4B).

**Figure 5:**
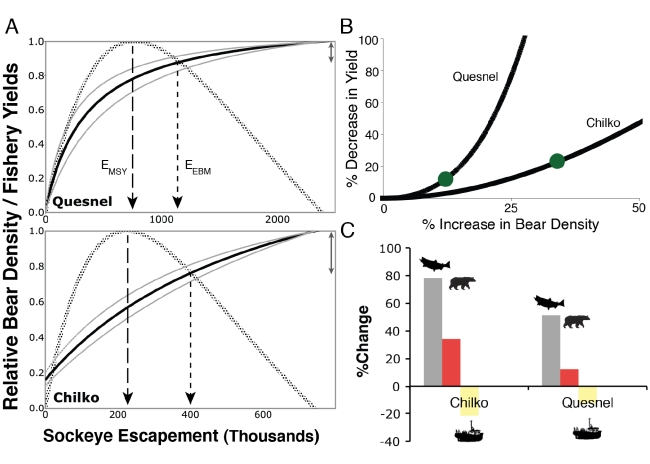
Using bears for ecosystem-based management in Chilko and Quesnel. (A) The relative bear density (solid) and relative fisheries yield (hatched) across a range of sockeye salmon escapements in Chilko and Quesnel (Fraser River) systems from British Columbia (BC), Canada. Ecosystem-based escapement goals, *E_EBM_*, occur where the curves meet, indicating that bears and fishery yields are equally reduced from their maxima (double-sided arrows). Increases in escapement from *E_MSY_* (maximum sustainable yield escapements; dashed arrows) to *E_EBM_* (dotted arrows) reduce harvests to some fraction of *MSY*. (B) Tradeoffs between loss in fisheries yield and increase in grizzly bear densities for escapements greater than those corresponding to MSY. Green dots indicate proposed ecosystem-based management escapements (*E_EBM_*) for each system. Reduction in fishery yields can result in substantial increases in bear density. (C) However, increased salmon allocations to bears (gray) under *E_EBM_* provide much higher nutrient subsidies to terrestrial and aquatic systems than either the percent increase in bear densities (red) or decrease in fishery yields (yellow), which we suggest is due to the shape of the stock-recruitment relationships.

For stocks with predictable stock-recruitment relationships and overcompensating density dependence (Chilko and Quesnel), we detect conflict between benefits to bears and benefits to fisheries. Across a range of escapements, expected fishery yields increase until escapements produce MSY and decline thereafter (relative fisheries yield [RFY] line in Figure 5A). In contrast, predicted bear densities increase monotonically and saturate as escapements increase (relative bear density [RBD] line in Figure 5A). In these interior systems of the Fraser River, where species other than sockeye contribute relatively little to total available salmon biomass, realizable bear densities are highly dependent on sockeye escapement (y-intercept of RBD in Figure 5A). Increasing escapement beyond *E_MSY_* leads to conflict between fishery yields and bear density, with the former decreasing and the latter increasing.

To aid in resolving such conflict in these systems and others, we provide here a straightforward EBFM decision-making framework. By scaling yields and bear densities relative to their maxima (that occur at *E_MSY_* and in the absence of fishing, respectively), we compare the dimensionless and commensurate values of *RFY* [15] and *RBD*. When *RFY* and *RBD* are equal, which is visualized at the intersection of *RFY* and *RBD* when plotted together (Figure 5A), equal relative costs are imposed on bears and fishers. We propose that this escapement level, which places equal social value to fisheries and the ecosystem, be termed “ecosystem-based management escapement”, or *E_EBM_*.

Managing at *E_EBM_*, rather than at *E_MSY_*, would impose considerable costs to fisheries. Losses in long-term yield are about 12% and 23% in the Quesnel and Chilko systems, respectively (Figure 5B and 5C). Based on 10-year average ex-vessel prices, lost revenues would be approximately C$680,000 and C$480,000 annually. These losses in yield would correspond to proportionally greater increases in escapement, however, nearing 50% in the Quesnel system and 80% in the Chilko run (Figure 5C).

These *E_EBM_* escapement levels, however, represent only one option within a continuum of ecosystem-harvest tradeoffs. We quantified these tradeoffs to assess losses in yield associated with increased bear densities as escapement varies above *E_MSY_* (Figure 5B). Costs to fisheries for increasing bear densities accrue slowly at first (low initial slope) and then accelerate.

## Discussion

Our goal here was to assess quantitatively the expected impact to fisheries and grizzly bears—a surrogate for salmon ecosystem function—if status quo management was adjusted to increase escapement across a range of contexts. We present a general framework that is flexible enough to address salmon management in systems that vary in escapement targets that themselves vary as a function of certainty in stock-recruitment relationships. In low certainty systems, managed at targets below estimated *E_MSY_*, the benefits to bears (and fisheries) of increased escapements can be assessed, but fishery yields are too uncertain beyond this level to assess accurately the tradeoffs. In relatively high certainty systems managed for MSY, we were able to evaluate the system-specific tradeoffs between the costs to humans in lost yield and the benefits of salmon escapement to bears (and the ecosystem) if escapements were to increase.

Any departure from current management would necessarily involve conflict between multiple competing objectives. Whereas forgoing yield for increased bear densities with escapements beyond *E_MSY_* in the Chilko and Quesnel systems represents obvious tradeoffs, others are more complex. For example, the expected increase in both bear density and fishery yield in the other four systems results in an apparent win-win situation where both the ecosystem and fisheries benefit from increasing escapement. However, high annual variability in recruitment could sometimes lead to a fishery closure if higher escapement targets committed to cannot be met. One way to avoid this is to increase upper escapement goals while retaining lower goals, which would continue to allow some fishing in low return years (as long as lower escapement goals are met) while allowing for increased escapement in other years. Retaining lower escapement goals may benefit subsistence fishers, who must harvest some fish each year but face restrictions if escapements are perceived to be too low. Finally, although we argue that the grizzly bear offers a sensible and attractive surrogate for salmon ecosystem function, additional ecosystem responses to different management options might instead be considered. For example, increasing net nutrient input into systems (e.g., [23]) or trophic (egg) subsidies to resident fishes (e.g., [24]) might also form reasonable and important ecosystem objectives. Similarly, minimizing the probability of years without harvests might form a desirable management objective; a quantitative evaluation of these tradeoffs might lead to very different escapement targets. In our system and others, multiple competing objectives like these increase complexity for managers, though relevant methods have been developed for decision-making (e.g., [25],[26]).

One utility of our approach is that it offers a quantitative method to evaluate how well various harvest options satisfy the MSC ecosystem criterion while accounting simultaneously for the potential economic costs to fishers. Our results suggest that low sockeye escapement is most detrimental to bears in systems where there is little biomass available from other salmon species. For example, because Nushagak has large runs of all five Pacific salmon species, salmon are expected to represent roughly 63% of bear diets even in the absence of sockeye (Figure 4A). In contrast, nearly no salmon other than sockeye is available in the Quesnel run. This makes consideration of ecosystem needs in salmon management particularly important for inland stocks, where abundant runs of pink (*O. gorbuscha*) and chum (*O. keta*) salmon are absent. Moreover, in all six systems, which have received MSC certification, the observation that bear densities can increase substantially with increased escapement from current management levels implies that fisheries compete with bears and other ecosystem recipients. This suggests that the “minimal ecosystem impact” criterion, currently satisfied with certification, might in fact require increased scrutiny. This might be particularly the case with the newly certified Fraser River sockeye; grizzly bears are provincially threatened in the Chilko and partially extirpated in the Quesnel system (Figure 2; [27]). Thus, the significant restrictions to bear population productivity we document as a result of conflict with fisheries are relevant to bear conservation.

Another utility of our approach, particularly when applied to systems with high certainty managed at MSY, is that it offers a novel conceptual and philosophical framework of conservation value. Although arbitrary, the escapement that imposes equal costs on bears and fisheries, *E_EBM_*, can serve as a starting point to guide what are likely to be contentious management decisions. Although provocative, we highlight that this target would provide greater benefit than expected; the additional sockeye escapement to bears (and the ecosystem) at *E_EBM_* relative to *E_MSY_* is greater than the penalty to fishers might suggest (Figure 5C). Such unexpectedly large contributions of salmon carcasses to broader ecosystem beneficiaries might form a good conservation investment. Compelling support for an “abundance matters” hypothesis is now emerging [28]; that is, while often site-specific, evidence is accumulating that suggests increased spawning density is associated with positive ecological responses across a broad array of taxa, including aquatic primary productivity [29], terrestrial vegetation growth [30],[31], invertebrate density [31], songbird density [32], and growth rates of resident fish (including juvenile salmon [33]), as well as other aquatic and terrestrial ecological processes [34]. Higher salmon escapement might also provide increased opportunities for salmon-based eco-tourism [28].

Adopting *E_EBM_* escapement goals using bears as an ecosystem surrogate has several additional desirable properties. First, implementing *E_EBM_* might be more politically robust than increasing escapements above *E_MSY_* by some arbitrary amount. Due to the saturating relationship between salmon biomass and bear density, harvests are not sacrificed in systems where bears can maintain high densities. Second, *E_EBM_* is environmentally robust. In systems with lower relative bear densities, moderate reductions in yield can translate to substantial gains for bears and ecosystems (Figure 5C). Third, this model, which makes tractable the complex cross-boundary interactions between salmon nutrients and multiple beneficiaries, reflects a quantifiable ecosystem approach to management. Implementation of this method by managers can be refined with a site-specific approach relating bear diets to salmon availability across years from focal populations, rather than across populations as we have done. Finally, recognizing that *E_EBM_* might not be socio-politically possible, our tradeoff curve approach (Figure 5B) allows estimation of costs and benefits associated with adjustments to escapement in either direction.

Applying our framework to other fisheries requires the following consideration. First, critical knowledge sets for focal non-target species should include not only their estimated population responses across a range of fish biomass, but also some distinguishing role the candidate species serves in the ecosystem (e.g., keystone function). Additionally, estimates of the costs to fisheries across a range of management options that depart from the status quo are critical. Moreover, selecting focal species of conservation concern to resource managers and the public might extend greater political will to any EBFM recommendation (see also [35]). Finally, we note that the principles of single-species fisheries management and EBFM depart conceptually and practically. The former focuses narrowly and almost exclusively on the exploitation of natural resources for humans, whereas EBFM is inclusive of all biodiversity, including humans. Our proposed EBFM targets, in which costs are equally borne by fisheries and bears (and by extension, the ecosystem), closely match the spirit of EBFM.

## Materials and Methods

We used a multi-stage analysis to predict how bear population density would respond to variation in spawning salmon abundance as influenced by harvest management. This involved first estimating a relationship between salmon abundance and salmon consumption by bears, and then linking this result to a known positive relationship between salmon consumption by bears and bear density.

### Salmon Abundance and Salmon Consumption by Bears

We used estimates of the proportion of salmon (including Kokanee) in the diet of bears from 18 grizzly bear population units (GBPUs) in BC, Canada, that were derived from stable isotope analysis [36]. These estimates were derived from hair, which grows throughout most of the annual activity period of bears. For these same GBPUs across the same period (1995–2003), we estimated the mean annual salmon biomass potentially available to bears (after interception by fisheries; the “escapement”). This involved using spatially explicit escapement data for all five species (pink, chum, coho, sockeye, and Chinook) to estimate the salmon returns in each of the watersheds captured by GBPUs (Figure 2C). We assigned a portion of these estimates to GBPUs based on the fraction of each watershed that intersects each GBPU. We converted salmon numbers to biomass, using average masses of each species and sex [37], assuming a 50∶50 ratio between sexes.

To determine how the availability of salmon biomass, *S* (kg/km^2^), influenced the proportion of salmon in grizzly bear diets, *D*(*S*), we fit a saturation curve using nonlinear least squares (Equation 1). Stable isotope data from grizzly bear hair sampled in the Columbia River basin, United States, during the late 1800s, when salmon were much more abundant, indicate that salmon can represent up to 90% of bear diets [38]. Several current bear populations consume more than 80% salmon [36], but—logically—we constrained consumption to values less than 100%. Accordingly, we fixed the asymptotic maximum consumption (i.e., the consumption when there are infinite salmon on the landscape) at 90% and used the data to fit the half-saturation parameter of the saturation curve. Robust estimation of the half-saturation parameter, and its confidence interval, is key because the 90% assumption will cancel in our analysis.

Percent salmon in diet, *D*(*S*), as a function of salmon biomass density, *S* (*kg/km^2^*), is given by
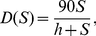
(1)where *h* is the half-saturation parameter that determines how quickly bear diets respond to salmon availability. We tested the derived relationship (Equation 1) at the watershed level (as opposed to population [i.e., GBPU] level) using escapement data from Rivers Inlet and Quesnel (BC) [22] and Ugashik and Egegik (Alaska) [39],[40] to estimate salmon consumption by bears (see Tables S1 and S2). The Rivers Inlet escapement and stable isotope data are from 1998 and 1999, when salmon were relatively rare due to an extremely poor sockeye run (Table S1). Note that we estimated biomass density by summing over escapements of all salmon species. We grouped Egegik and Ugashik watersheds (Figure 2) and compared predicted dietary salmon (Ugashik: 67.2%, Egegik: 76.6%, average: 71.9%) with the average estimates from stable isotope data, also from hair, collected in the associated Alaska Game Management Units 9B, 9C, and 9D (71%, 73%, and 73% dietary salmon, respectively, average of 72.3%) [36]. Because the Quesnel sockeye run is cyclic, we used the median, rather than mean, escapement since it is a more robust approximation of inter-annual biomass availability.

### Fishery Yields

We determined the expected salmon harvest (run size minus escapement) using standard Ricker stock-recruitment models (Figure 3), which are well suited to characterize overcompensating density dependence [39]. They are also conservative in favor of fisheries because yields decline more quickly with increased escapement than if Beaverton-Holt dynamics are assumed.

The size of the recruited salmon population *R*, when the spawning population is *E*, is given by
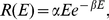
(2)and yield is simply recruitment minus escapement.

The escapement that maximizes long-term sustainable yield is *E_MSY_*, which we determined graphically based on the best-fit parameters. However, it is often difficult to estimate *E_MSY_* because many stock-recruitment relationships are fraught with uncertainty in parameter estimates and even uncertainty over whether the stock-recruitment relationship is appropriate to describe the dynamics of the fishery. As a result, fisheries with adequate stock-recruitment data can be managed by targeting a biologically based escapement of *E_MSY_* (called a “biological escapement goal”). Other fisheries are managed between lower and upper target escapements that have provided adequate yield in the past (called a “sustainable escapement goal”), but this escapement range is not necessarily optimal (i.e., maximizing long-term yield). Because our goal was to determine how departures from status quo management impact bears and ecosystems, we conducted distinct analyses for stocks managed at *E_MSY_* and those managed for a range of target escapements that were generally below estimates of *E_MSY_* as determined by stock-recruitment relationships.

For fisheries managed at *E_MSY_*, the relative fishery yield (*RFY*) achieved with escapement *E* relative to the maximum yield is
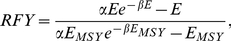
(3)which is a measure of the proportion of yield achieved by the fishery when escapement is *E* compared with when yields are maximized at *E_MSY_*. For fisheries managed for a range of target escapements, we used the same functional form but with the lower target escapement as our management baseline rather than *E_MSY_* (Figure 4B).

### Linking Salmon Consumption by Bears to Bear Density

We consider the bear density at a particular escapement relative to the bear density at the stock-specific maximum escapement (i.e., no fishery). The escapement in the absence of the fishery, *E_m_*, is the escapement at the steady state (i.e., where recruitment and escapement are equal) of the Ricker stock-recruitment model,
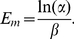
(4)


However, for fisheries without adequate certainty in stock recruitment data to estimate *E_m_*, we use the maximum observed escapement (Figure 3) instead. The maximum observed escapement in these stocks is well below estimates of *E_m_* from stock-recruitment relationships, which suggests that our projections of impacts of fisheries on bear populations are conservative. We estimated the expected bear density for a given level of escapement relative to the expected bear density with the maximum escapement (*E_m_*; Equation 4 or maximum observed escapement). Bear density, *B*, was estimated by linking Equation 1 with a known linear relationship between percent meat in diet and bear density [10], but we assumed a zero intercept, which is conservative in favor of fisheries because some meat is likely necessary to sustain even the smallest bear density. Note that we assumed all meat consumed in coastal populations was derived from salmon, a reasonable assumption based on data from multiple populations [36]. The bear density for a given escapement is thus
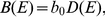
(5)where *b*_0_ determines how quickly bear densities increase with dietary salmon. Because bear densities increase linearly, *b*_0_ cancels when determining relative bear density so that our results depend only on the assumption of linearity and are not dependent on any particular slope from Equation 5. Although in practice bear densities are limited by bottom-up (i.e., salmon) and top-down (i.e., hunting) forces, bottom-up forces influence population productivity and potential bear densities in the absence of killing by humans [41].

Percent salmon in diet (Equation 1) saturates with salmon availability,
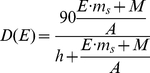
(6)where *m_s_* is the mean mass (kg) of an individual sockeye, *M* is an estimate of the biomass of all other salmon species present in each system, and *A* is the area (km^2^) of the watersheds that contains each salmon stock (Table S2). To estimate *M*, we used target escapement goals when they existed (mean of lower and upper goal) [39]; if not, we used average escapements from 1999 to 2008. For runs with neither escapement targets nor data, we used harvest to approximate escapement by assuming a 50% harvest rate [40] (see Table S2 for stock- and species-specific data sources).

The relative bear density, *RBD*, can be written by combining Equations 5 and 6 as
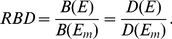
(7)Plugging Equation 6 into Equation 7 and simplifying, *RBD* becomes
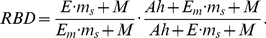
(8)The relative fisheries yield, *RFY*, and the relative bear density, *RBD*, are now both dimensionless and commensurate values that can be directly compared.

### Percent Change in Yields and Bear Densities in Systems with High Uncertainty in Stock-Recruitment Relationships

For stocks with high uncertainty, *E_MSY_* and *E_m_* could not be reliably estimated. Moreover, current management practice in these systems targets a range of escapements, bounded by lower and upper goals, rather than *E_MSY_* level escapements. For these stocks we calculated percent changes in bear densities and fisheries yields when increasing from lower escapement goals to upper goals and to *E_MSY_* (Figure 4B). To do this, we followed the same functional form as for *RFY* and *RBD* (Equations 3 and 8), but used the lower escapement goal as our baseline rather than *E_m_* and *E_MSY_*. Thus, rather than assess how bear densities and fishery yields compare to their system-specific maxima, we assessed how they are expected to respond to variation in the current management regime (i.e., from current lower to upper escapement goals), as well as how they are expected to respond when moving from lower escapement goals to predicted *E_MSY_*.

## Supporting Information

Table S1 **The biomass density (kg/km^2^) of each salmon species used to compare predicted to actual percent salmon in bear diets.** For Rivers Inlet, pink and chum escapements were higher during the years when sockeye were not being fished. We used the median, rather than mean, sockeye escapement when calculating biomass for Quesnel because this stock is cyclic and the median is a more robust estimate of biomass availability. All other biomass density estimates are consistent with Table S2.
(DOC)

Table S2 **The biomass density (kg/km^2^) of each non-sockeye salmon species and escapements (in thousands) for the six sockeye stocks we consider.** Biomass data come from the ^A^mean of lower and upper escapement goal from the 2009 Bristol Bay Escapement Review [39], ^B^ mean 1999–2008 harvests from the 2009 Bristol Bay Management Report, assuming 50% harvest rate [40], ^C^mean escapement 1999–2008 from Department of Fisheries and Oceans Canada spawning escapement database (unpublished data), ^D^MSY escapements calculated with stock recruitment models [42].
(DOC)

## Abbreviations

BC: British Columbia
EBFM: ecosystem-based fisheries management
GBPU: grizzly bear population unit
MSC: Marine Stewardship Council
MSY: maximum sustainable yield
RBD: relative bear density
RFY: relative fisheries yield
