# Supplementary material for: Using Grizzly Bears to Assess Harvest-Ecosystem Tradeoffs in Salmon Fisheries
Source: PLoS Biol. 2012 Apr 10;10(4):e1001303. doi: 10.1371/journal.pbio.1001303 (PMC3323506; doi:10.1371/journal.pbio.1001303)
Supplement: Table S1 — The biomass density (kg/km2) of each salmon species used to compare predicted to actual percent salmon in bear diets. For Rivers Inlet, pink and chum escapements were higher during the years when sockeye were not being fished. We used the median, rather than mean, sockeye escapement when calculating biomass for Quesnel because this stock is cyclic and the median is a more robust estimate of biomass availability. All other biomass density estimates are consistent with Table S2. (DOC) [file pbio.1001303.s001.doc]

**Table S1**

| **Stock** | **Years** | **Pink** | **Chum** | **Chinook** | **Coho** | **Sockeye** |
| --- | --- | --- | --- | --- | --- | --- |
| **Rivers Inlet Collapse** | **1998-1999** | **67.33** | 21.74 | 0.00 | 0.00 | 27.24 |
| **Quesnel** | **1995-2003** | 0.00 | 0.00 | 8.18 | 0.06 | 34.84 |
| **Ugashik** | **1995-2003** | 0.00 | 0.00 | 1.19 | 0.28 | 205.70 |
| **Egegik** | **1995-2003** | 0.00 | 28.11 | 1.52 | 0.72 | 199.03 |
