# Supplementary material for: Using Grizzly Bears to Assess Harvest-Ecosystem Tradeoffs in Salmon Fisheries
Source: PLoS Biol. 2012 Apr 10;10(4):e1001303. doi: 10.1371/journal.pbio.1001303 (PMC3323506; doi:10.1371/journal.pbio.1001303)
Supplement: Table S2 — The biomass density (kg/km2) of each non-sockeye salmon species and escapements (in thousands) for the six sockeye stocks we consider. Biomass data come from the Amean of lower and upper escapement goal from the 2009 Bristol Bay Escapement Review [39], B mean 1999–2008 harvests from the 2009 Bristol Bay Management Report, assuming 50% harvest rate [40], Cmean escapement 1999–2008 from Department of Fisheries and Oceans Canada spawning escapement database (unpublished data), DMSY escapements calculated with stock recruitment models [42]. (DOC) [file pbio.1001303.s002.doc]

**Table S2**

| **Stoc** | **Area**  **(km^2^)** | **Pink** | **Chum** | **Chinook** | **Coho** | **Sockeye**  **Elow** | **Sockeye**  **Eup** | **Sockeye**  ***E_MSY_*** | **Sockeye**  ***E_m_*** | **Sockeye**  ***E_EBM_*** |
| --- | --- | --- | --- | --- | --- | --- | --- | --- | --- | --- |
| **Rivers Inlet** | **8910** | 41.85^D^ | 9.77 ^D^ | 0.00 | 1.9 ^D^ | **200** | **610** | **1150** | **2460** | **1445** |
| **Chilko** | **19548** | 0.00 | 0.00 | 8.18 ^C^ | 0.06 ^C^ | **---** | **---** | **224** | **742** | **400** |
| **Quesnel** | **12009** | 0.00 | 0.00 | 1.19 ^C^ | 0.28 ^C^ | **---** | **---** | **763** | **2380** | **1155** |
| **Ugashik** | **11157** | 0.00 ^B^ | 28.11^B^ | 1.52 ^B^ | 0.72 ^B^ | **500** | **1200** | **2145** | **5140** | **2715** |
| **Egegik** | **7185** | 0.06 ^B^ | 37.56 ^B^ | 0.85 ^A^ | 6.90 ^B^ | **800** | **1400** | **6305** | **16405** | **7285** |
| **Nushagak** | **20794** | 53.14 ^A^ | 32.89 | 39.24 ^A^ | 11.54 ^A^ | **340** | **760** | **749** | **1930** | **1005** |
